# Immunosuppressive Drugs Affect High-Mannose/Hybrid N-Glycans on Human Allostimulated Leukocytes

**DOI:** 10.1155/2015/324980

**Published:** 2015-08-03

**Authors:** Ewa Pocheć, Katarzyna Bocian, Marta Ząbczyńska, Grażyna Korczak-Kowalska, Anna Lityńska

**Affiliations:** ^1^Department of Glycoconjugate Biochemistry, Institute of Zoology, Faculty of Biology and Earth Science, Jagiellonian University, Gronostajowa 9, 30-387 Krakow, Poland; ^2^Department of Immunology, Institute of Zoology, Faculty of Biology, University of Warsaw, Miecznikowa 1, 02-096 Warsaw, Poland; ^3^Department of Clinical Immunology, Transplantation Institute, Medical University of Warsaw, Nowogrodzka 59, 02-006 Warsaw, Poland

## Abstract

N-glycosylation plays an important role in the majority of physiological and pathological processes occurring in the immune system. Alteration of the type and abundance of glycans is an element of lymphocyte differentiation; it is also common in the development of immune-mediated inflammatory diseases. The N-glycosylation process is very sensitive to different environmental agents, among them the pharmacological environment of immunosuppressive drugs. Some results show that high-mannose oligosaccharides have the ability to suppress different stages of the immune response. We evaluated the effects of cyclosporin A (CsA) and rapamycin (Rapa) on high-mannose/hybrid-type glycosylation in human leukocytes activated in a two-way mixed leukocyte reaction (MLR). CsA significantly reduced the number of leukocytes covered by high-mannose/hybrid N-glycans, and the synergistic action of CsA and Rapa led to an increase of these structures on the remaining leukocytes. This is the first study indicating that *β*1 and *β*3 integrins bearing high-mannose/hybrid structures are affected by Rapa and CsA. Rapa taken separately and together with CsA changed the expression of *β*1 and *β*3 integrins and, by regulating the protein amount, increased the oligomannose/hybrid-type N-glycosylation on the leukocyte surface. We suggest that the changes in the glycosylation profile of leukocytes may promote the development of tolerance in transplantation.

## 1. Introduction

Most cell surface and secreted proteins in the immune system are N-glycosylated [1–4]. N-glycans added to the special protein sequence (Asn-X-Ser/Thr) during the posttranslational process form a large and important part of glycoprotein molecules; the oligosaccharide component reaches up to even 50% of glycoprotein molecular mass [5]. N-glycoproteins contain three different oligosaccharide structures: high-mannose, hybrid, and complex-type [6]. High-mannose glycans have been considered to be incomplete products of the N-glycosylation synthesis pathway, evolutionarily older than complex-type structures. They are thought to be typical for simpler organisms like yeast or fungi [5, 7, 8], but some studies have shown that these oligosaccharide structures are also abundant in mammalian cells and are no less important than completely processed complex-type sugar chains [9, 10]. Recognition of high-mannose structures by mannose receptors in macrophages and dendritic cells is critical to the innate immune response responsible for elimination of bacteria and viruses and for initiation of organ-specific autoimmunity [11, 12]. Other work established that mannose-rich oligosaccharides can suppress the immune response [13], ligand binding [14], and intracellular signal transduction [15].

Integrins are transmembrane receptors which mediate adhesive events critical to an effective immune response [16–19]. Leukocytes express a broad range of *αβ* integrin heterodimers (at least 12) [17, 19]. The integrin repertoire on the leukocyte surface depends on the stage of leukocyte activation and is regulated by cytokines, chemokines, and other adhesion receptors [20]. The functions of leukocytes rely mostly on integrins belonging to the *β*2 family but also to the *β*1 family [16], and *β*3 integrins are important to leukocyte biology as well [19, 20]. They are also called very late antigens (VLA), because some integrins appear on the cell surface days or even weeks after leukocyte activation [21]. Integrins take part in the formation of immunological synapses [21, 22], which serve immune cell communication [23]. These adhesion proteins are needed for leukocytes to move from the blood into peripheral tissues [20, 24] and they mediate leukocyte interactions with endothelial cells and extracellular matrix proteins (ECM) [20]. The most important integrins for leukocyte activation and migration are LFA-1 (*α*L*β*2), *α*4*β*1 (VLA-4), and *α*4*β*7 [25–27]. N-glycosylation of integrins, which are the highly N-glycosylated proteins, plays a crucial role in their functioning [28].

The proper functioning of immune cells in the response to alloantigens depends strongly on glycosylation of surface receptors [4]. Leukocytes modify cell interactions during each stage of the immune response by regulating the type and abundance of glycans; differentiation of leukocytes is also dependent on their glycosylation [8, 29–31]. The N-glycosylation process is very sensitive to different environmental agents and to the pathological conditions of immune diseases [29, 32, 33]. In transplantation, strong inflammatory signals are induced in the recipient, which need to be controlled through administration of immunosuppressive drugs [34, 35].

We posited that the pharmacological environment of immunosuppressive drugs may modulate leukocyte glycosylation. To the best of our knowledge there are no published studies evaluating the effects of cyclosporin A (CsA; inhibitor of calcineurin) and rapamycin (Rapa; inhibitor of mammalian target of rapamycin mTOR) on human immune cell glycosylation. In this study we assessed effects of these drugs on leukocyte glycosylation. These are immunosuppressive agents commonly used to inhibit leukocyte activation during a rapid immune response. We found that CsA significantly reduces the number of leukocytes covered with high-mannose/hybrid N-glycans, while the synergistic action of CsA and Rapa leads to an increase of these structures on the remaining leukocytes. We demonstrated, for the first time, that *β*1 and *β*3 integrins bearing high-mannose/hybrid structures are influenced by Rapa and CsA. Rapa taken separately and together with CsA significantly altered the expression of *β*1 and *β*3 integrins, and this caused an increase in the amount of oligomannose/hybrid N-glycans on leukocyte surfaces.

## 2. Materials and Methods

### 2.1. Materials

Peripheral blood samples were obtained from healthy volunteers aged 18–60 years. Gradisol L was provided by Aqua Medica (Łódź, Poland). RPMI1640 medium was purchased from Biomed, fetal calf serum (FCS), L-glutamine, and N-2-hydroxyethyl-piperazine-N′-2-ethanesulphonic acid (HEPES) from Gibco, and gentamicin from Biochemie. Rapamycin and cyclosporin A were obtained from Wyeth-Lederle and Novartis Pharma, respectively. Laemmli sample buffer (LSB, 161-0737) and *β*-mercaptoethanol (*β*-ME) were from Bio-Rad and RIPA buffer (89900) and PageRuler Prestained Protein Ladder (26616) from Thermo Scientific. Biotinylated* Galanthus nivalis* agglutinin (GNA, B-1246), agarose-bound GNA (AL-1243), agarose-bound streptavidin (SA-5010), and Carbo-Free Blocking Solution were obtained from Vector Lab. Mouse antibody against *β*1 integrin subunit (MAB2251, clone B3B11), rabbit against *β*3 subunit (AB1932), and alkaline phosphatase- (AP-) conjugated sheep anti-rabbit (AP322A) were obtained from Millipore. Rabbit polyclonal anti-GAPDH (G9545), AP-conjugated goat anti-mouse (084K4861) antibody, AP-conjugated ExtrAvidin (E2636), FITC-conjugated ExtrAvidin (E2761), Coomassie Brilliant Blue G (B2025), protease inhibitor cocktail (P2714), and trypan blue (T8154) were purchased from Sigma-Aldrich. Horseradish peroxidase- (HRP-) conjugated horse anti-mouse (7076S) and sheep anti-rabbit (70742) antibody were obtained from Cell Signaling Technology. Substrate for AP, nitro blue tetrazolium chloride (NBT) and 5-bromo-4-chloro-3-indolyl-phosphate (BCIP), and recombinant endo-*β*-N-acetylglucosaminidase H (Endo H) from* Streptomyces plicatus* (11088726001) were purchased from Roche. Western Bright Sirius chemiluminescent HRP substrate (K-12043-D10) was obtained from Advansta.

### 2.2. Mixed Leukocyte Reaction

Peripheral blood mononuclear cells (PBMCs) were isolated from heparinized venous blood by density-gradient centrifugation over Gradisol L. PBMCs were resuspended in RPMI1640 medium supplemented with 10% FCS, 20 mM L-glutamine, 10 *μ*g/mL gentamicin, and 1 M HEPES and counted in a hemocytometer chamber using trypan blue dye. Cells from two healthy donors (1 × 10^6^ per 1 mL medium) were mixed 1 : 1 for the two-way mixed leukocyte reaction (MLR). The cells were cultured in 24-well plates at 37°C for 6 days in a CO_2_ incubator (Lab Line Instruments) in the presence of CsA (200 ng per 1 mL medium), Rapa (20 ng per 1 mL medium), and both drugs given together (150 ng CsA and 12 ng Rapa per 1 mL medium). The choice of immunosuppressive drug doses was based on earlier studies [36, 37].

### 2.3. Flow Cytometric Analysis

The cells were harvested from MLR cell culture and washed twice in cold PBS. All the steps were performed on ice. Cells were incubated with biotinylated GNA (1 : 100) for 30 min at RT, centrifuged (1800 rpm, 5 min, 4°C), and washed in PBS. Then the cells were incubated with FITC-conjugated ExtrAvidin (1 : 100) for 30 min at RT and centrifuged under the same conditions as previously. After washing in PBS, fluorescence was measured by flow cytometry in a FACSCalibur (BD Biosciences) using Cell Quest software (BD Biosciences).

### 2.4. GNA Precipitation

Cell lysate proteins (500 *μ*g) were incubated with 25 *μ*L agarose-bound* Galanthus nivalis* lectin (GNA) in HEPES buffer (10 mM HEPES, pH 7.5, containing 150 mM NaCl) and incubated overnight at 4°C. After centrifugation (14,000 rpm, 2 min, RT) the supernatants from the GNA-treated protein extracts were collected. The precipitates were washed three times in HEPES buffer and once in PBS. Glycoproteins were released from the glycoprotein-lectin-agarose complexes by boiling 10 min in 25 *μ*L LSB containing *β*-ME at 100°C for 10 min. After final centrifugation the supernatants containing GNA-positive glycoproteins were collected and destined for SDS-PAGE separation followed by MS/MS analysis.

### 2.5. Lectin Blotting

GNA precipitates, the supernatant collected after GNA precipitation, and cell lysate proteins were separated on 10% SDS-PAGE under reducing conditions and then transferred to a PVDF membrane. To detect proteins carrying high-mannose N-oligosaccharides, the PVDF blots were blocked in Carbo-Free Blocking Solution overnight at 4°C. After washing three times in TBST and once in TBS with 1 mM MgCl_2_, 1 mM MnCl_2_, and 1 mM CaCl_2_, the membranes were incubated with biotinylated GNA lectin (1 : 4000) in TBS with the ions for 1 h at RT. After further washing in TBST the membranes were incubated for 1 h at RT with AP-conjugated ExtrAvidin (1 : 4000). After final washing, the GNA-positive proteins were visualized using NBT/BCIP solution as substrate for AP.

### 2.6. MS/MS Analysis

GNA precipitates (20 *μ*L from 25 *μ*L total volume) were separated by SDS-PAGE. The gel was fixed with 7% acetic acid in 40% methanol (v/v) for 30 min and stained with Coomassie Brilliant Blue for 2 h. The gel bands corresponding to the GNA-positive proteins (separated on the same gel but destined for lectin reaction on a PVDF membrane; the 5 *μ*L remainder from 25 *μ*L total volume) were excised, subjected to trypsin digestion, and analyzed by tandem mass spectrometry MS/MS in the Mass Spectrometry Laboratory of the Institute of Biochemistry and Biophysics, Polish Academy of Sciences (Warsaw, Poland). Proteins were identified by matching the peptides with the Sprot nonredundant database (547357 sequences/194874700 residues) with a* Homo sapiens* filter (20274 sequences) from the Mascot Distiller software (version 2.4.2.0, MatrixScience). Scores higher than 46 were considered to be significant (*p* < 0.0005).

### 2.7. Endo H Digestion

The content of high-mannose and hybrid glycans in integrin subunits was determined using endo-*β*-N-acetylglucosaminidase H from* Streptomyces plicatus*. The cell glycoproteins (15 *μ*g) were suspended in sodium phosphate buffer (pH 5.5) to 50 mM final concentration. Protein denaturation was carried out in the presence of 0.2% SDS and 1 M *β*-ME for 3 min. After cooling to RT, 25 mU Endo H was added and the samples were incubated overnight at 37°C. The reaction was stopped by heating at 100°C for 10 min. Both digested and nondigested samples were boiled in LSB with *β*-ME prior to SDS-PAGE on 10% polyacrylamide gels.

### 2.8. Immunoblotting

Equal amounts of proteins in LSB with *β*-ME (15 *μ*g) were separated on 10% polyacrylamide gel and transferred to a PVDF membrane. After blocking in 1% BSA in TBST, integrin subunits were incubated for 1 h at RT with anti-*β*1 and anti-*β*3 antibody diluted 1 : 1000 in 1% BSA in TBST. Rabbit anti-GAPDH IgG was diluted 1 : 4000 and incubated on a membrane in the same conditions. Then anti-mouse IgG and anti-rabbit IgG were applied in a 1 : 4000 dilution and incubated at RT for 1 h. Integrin subunits were visualized by chemiluminescence in the GeneGnome Imaging System (Syngen) or with the colorimetric reaction by incubating the membranes with BCIP/NBT substrate for AP. The oligomannose/hybrid N-glycan amounts were calculated based on loss of molecular mass after Endo H digestion using UVImap Image Quantification software (UVItec).

## 3. Results

### 3.1. CsA Reduces GNA-Positive Cell Number, but the Synergistic Action of CsA and Rapa Increases the Amount of GNA-Positive Glycoproteins

**Figure 1:**
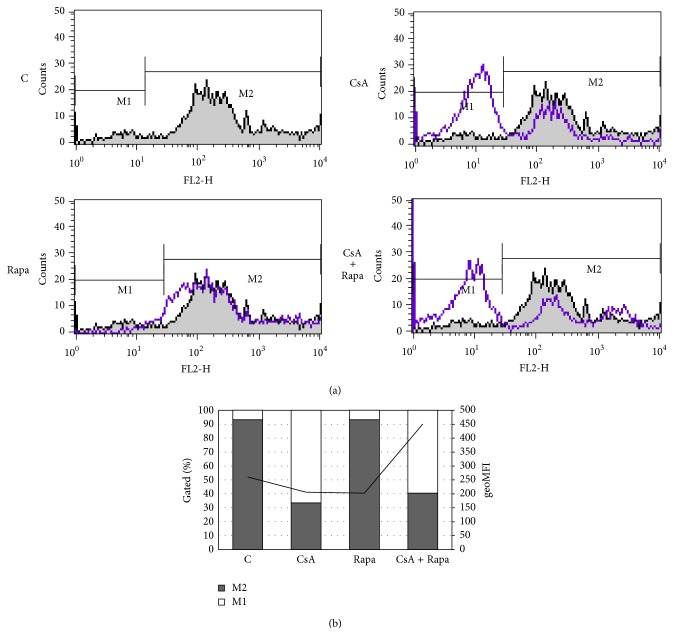
Immunosuppressive drugs alter the surface expression of high-mannose/hybrid N-glycans. Peripheral blood mononuclear cells were cultured for 6 days in the presence of CsA (200 ng per 1 mL medium), Rapa (20 ng per 1 mL medium), and the combination of CsA (150 ng per 1 mL medium) and Rapa (12 ng per 1 mL medium) in a two-way mixed leukocyte reaction (MLR). Immunosuppressive drug-treated and control cells were stained with biotinylated GNA (1 : 100), followed by incubation with FITC-conjugated ExtrAvidin. M2 region corresponds to GNA-positive leukocytes after allostimulation. Fluorescence was measured using a FACSCalibur flow cytometer (BD Biosciences). C: untreated cells, CsA: cyclosporin A, and Rapa: rapamycin.

Glycosylation of alloantigen-activated PBMCs from two individuals mixed together in two-way MLR cell culture was analyzed using FITC-labeled* Galanthus nivalis* agglutinin (GNA) in flow cytometry. We found that the number of leukocytes covered by oligomannose/hybrid-type N-glycans decreased dramatically in the presence of CsA applied alone or with Rapa (Figure 1). Mean fluorescence intensity (MFI) was reduced slightly by the drugs given separately but the combination of both drugs significantly raised the MFI value. CsA was responsible for removal of high-mannose/hybrid N-glycans from the surface of over 60% of the leukocytes, but the synergistic effect of both immunosuppressive drugs markedly increased the amount of oligomannose/hybrid-type N-glycans on the remaining cells.

### 3.2. Glycoproteins Bearing High-Mannose/Hybrid Structures Are Affected by CsA and Rapa

**Figure 2:**
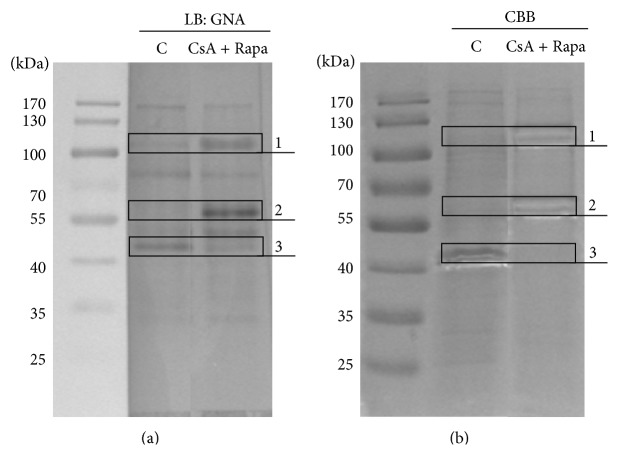
Immunosuppressive drugs change the intensity of bands containing GNA-positive proteins. Peripheral blood mononuclear cells were cultured for 6 days in the presence of the combination of CsA (150 ng per 1 mL medium) and Rapa (12 ng per 1 mL medium) in a two-way mixed leukocyte reaction (MLR) and then lysed in RIPA buffer. Whole-cell lysate proteins (500 *μ*g) were precipitated with 25 *μ*L agarose-bound* Galanthus nivalis* lectin (GNA). The captured glycoproteins were recovered by boiling in Laemmli sample buffer with *β*-ME and resolved by 10% SDS-PAGE under reducing conditions. One-fifth of the GNA precipitate was destined for Western blotting (WB) and after probing with biotinylated-GNA was visualized by AP colorimetric reaction (a). The remaining four-fifths of the precipitate, after resolving on the same gel, were stained with Coomassie Brilliant Blue (CBB) and the bands were excised for MS/MS analysis (b). Molecular weight of proteins was assigned using a PageRuler Prestained Protein Ladder (Thermo Scientific, 26616). LP: lectin precipitation, C: untreated cells, CsA: cyclosporin A, and Rapa: rapamycin.

Lectin blotting (LB) with biotinylated GNA disclosed changes in the oligomannose/hybrid-type glycosylation of some proteins in samples with CsA given together with Rapa; the reaction was more intense in bands 1 and 2, while reduction of GNA binding was seen in band 3 (Figure 2(a)). Based on the GNA reaction, bands found to have been altered in intensity by immunosuppressive agents were excised from the gels (Figure 2(b)) and their content was analyzed using MS/MS. This assay showed 62 possible proteins in band 1, 151 in band 2, and 126 in band 3. The ten identified proteins with the highest scores for each band are listed in Table 1. Among them are transmembrane proteins (*β* integrins, HLA class I histocompatibility antigen, LAMPs) and cytoplasmic proteins (enzymes of various metabolic pathways, coagulation factors). The molecular mass of the identified monomeric glycoproteins, calculated based on the amino acid sequences, was lower than the mass determined in SDS-PAGE, due to the presence of N-glycan components. The theoretical molecular weight of multimeric glycoproteins is higher than that determined based on Protein Ladder standards, because the samples were resolved in reducing conditions.

### 3.3. Expression of *β*1 Precursor and *β*3 Integrin Subunits Is Changed by CsA and Rapa

**Figure 3:**
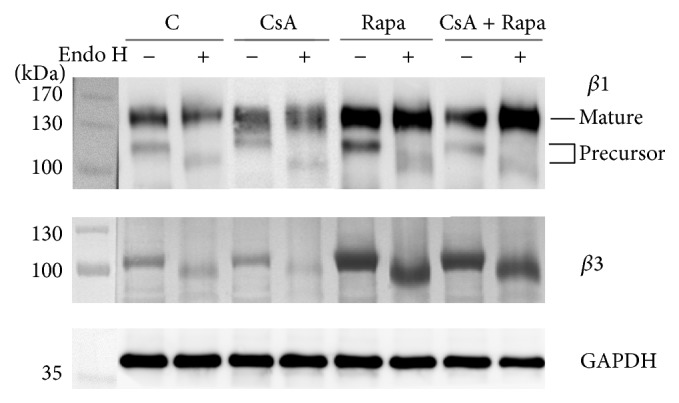
Rapamycin (Rapa) and the combination of cyclosporin A (CsA) and Rapa changes *β*1 and *β*3 integrin expression on MLR-activated cells. Peripheral blood mononuclear cells were cultured for 6 days in the presence of CsA (200 ng per 1 mL medium), Rapa (20 ng per 1 mL medium), and the combination of CsA (150 ng per 1 mL medium) and Rapa (12 ng per 1 mL medium) in a two-way mixed leukocyte reaction (MLR) and then lysed in RIPA buffer. Protein extracts (15 *μ*g) were digested with endo-*β*-N-acetylglucosaminidase H (Endo H) from* Streptomyces plicatus*, SDS-PAGE-separated on 10% gel under reducing conditions, electroblotted onto a PVDF membrane, and probed with specific primary antibodies: mouse monoclonal anti-*β*1 (Chemicon, MAB2251, clone B3B11) and rabbit polyclonal anti-*β*3 (Chemicon, AB1932). Antibody-bound integrins were visualized by chemiluminescence. GAPDH was the endogenous control. C: untreated cells.

**Figure 4:**
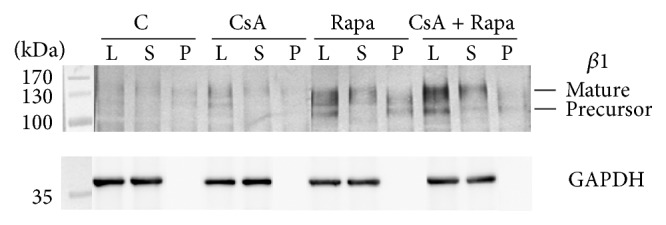
High-mannose/hybrid-type glycans are present mainly on premature *β*1 integrin subunit. Cell lysate (L), glycoproteins precipitated with GNA-agarose (P), and supernatant collected after precipitation (S), containing proteins not recognized by GNA, were resolved on 10% SDS-PAGE gel under reducing conditions, electrotransferred to a PVDF membrane and destined for *β*1 integrin subunit immunodetection. GAPDH was the endogenous control. C: untreated cells, CsA: cyclosporin A, and Rapa: rapamycin.

In further analyses we focused on the *β* integrin subunits identified in the band 1 to verify the results obtained by MS/MS analysis. Immunodetection of the *β*1 subunit after releasing the high-mannose/hybrid-type N-glycans with Endo H revealed that the *β*1 precursor, with lower molecular mass, mainly carries these types of N-glycans (Figure 3), but the mobility shift in the control and immunosuppressive drug-treated MLR cells was comparable. Comparable loss of molecular weight between the control and tested cells after Endo H digestion was also found in the *β*3 subunit. We observed, however, a significant increase of the intensity of the *β*1 (both forms) and *β*3 subunits in MLR cells cultured in the presence of Rapa alone or combined with CsA. GNA precipitation confirmed the results from Endo H digestion of the *β*1 subunit (Figure 4). GNA recognized mainly the *β*1 precursor form; the mature subunit remained unprecipitated in supernatants. Here we also observed higher intensity of the *β*1 subunit in MLR treated with Rapa and the combination of both drugs. These results indicate that high-mannose/hybrid-type glycosylation did not change on a single *β* integrin molecule but that the increased binding of GNA resulted from enhancement of *β* integrin expression (Figures 3 and 4) and that it contributed to the overall increase of high-mannose/hybrid-type glycans on the cell surface in the presence of CsA and Rapa (Figure 1).

## 4. Discussion

It is well documented that immunosuppressive drugs, designed to inhibit activation of the immune response [34], act by changing the expression of many proteins on immune cells [38–40], including integrins [41–44], but there are only few studies showing that they influence protein glycosylation [15, 45, 46]. For this reason we focused on determining the effects of the immunosuppressive drugs CsA and Rapa on N-glycosylation of leukocytes alloactivated* in vitro*. Flow cytometric analysis showed that CsA downregulated the amount of GNA-positive leukocytes, but both drugs acting synergistically increased the surface expression of high-mannose/hybrid-type N-glycans on the remaining leukocytes (Figure 1). Lectin blotting confirmed the enhancement of the reaction with GNA lectin of some SDS-PAGE-resolved glycoproteins (Figure 2(a)). The synergistic effects of CsA and Rapa, different from those of one-drug treatment, result from the various molecular mechanisms of these drugs [40]. They act at different stages of the T cell cycle: CsA at the G0 phase of the T lymphocyte cell cycle, and Rapa later at the transition from the G1 to the S phase. CsA interacts with calcineurin to block IL-2 gene transcription and then to inhibit T-cell proliferation, leading to decreased IL-2 production and secretion by T cells. Rapa blocks the intracellular signaling trigger by binding IL-2 to its receptor, thereby inhibiting T-cell responses to cytokines [38, 40, 47]. Most protocols use a combination of agents for induction and maintenance of immunosuppression to improve patient survival and graft survival rates and to reduce side effects [47].

In a proteomic study, Lee et al. [38] showed that most of the altered proteins from human T lymphocytes treated with CsA and a polysaccharopeptide have functional significance in protein degradation, the antioxidant pathway, energy metabolism, and immune cell regulation. Our MS/MS analysis revealed that among the human leukocyte glycoproteins affected by CsA and Rapa are HLA class I histocompatibility antigens (decreased reaction with GNA), enzymes of various metabolic pathways, coagulation factors, the adhesion protein LAMP1, and integrins of the *β*1 and *β*3 subfamilies (increased reaction with GNA) (Table 1). In further analyses we focused on the *β*1 and *β*3 integrin subunits. Immunodetection of *β*1 and *β*3 subunits after Endo H digestion showed that the more intense reaction with GNA in Rapa- and Rapa/CsA-treated leukocytes was not the result of changed oligomannose/hybrid glycosylation on these proteins, but rather increased expression of the integrins (Figure 3). Immunosuppressive agents usually act by decreasing protein expression, which leads to attenuation of the immune response and induces a state of tolerance [40]. From previous results it is known that the changes in integrin expression caused by immunosuppressive drugs or blocking antibodies depend on the target cells and on the type and even the dose of agents. In a recent study, Zal et al. [44] observed reduction of *α*2 and *β*1 integrin expression on kidney fibroblasts from CsA-treated rats. Sarnacki et al. [27] found that anti-LFA-1 protects against MHC-incompatible graft rejection of fetal small bowel grafts transplanted into mice, making this integrin a potential target for immunosuppression in intestinal transplantation. In turn, the effect of the novel immunosuppressive drug mycophenolate mofetil (MMF) on tumor cells was dose- and cell line-dependent. In kidney carcinoma Caki I cells and pancreatic carcinoma DanG cells treated with 0.1 *μ*M and 1 *μ*M MMF, the expression of integrins of the *β*1 subfamily (*α*1*β*1, *α*2*β*1, *α*3*β*1, *α*4*β*1, *α*5*β*1, and *α*6*β*1) was downregulated; in colonic adenocarcinoma HT-29, *α*3*β*1, and *α*6*β*1 integrins were upregulated in the presence of 1 *μ*M MMF; and in prostate carcinoma DU-145 most of the analyzed integrins (*α*1*β*1, *α*2*β*1, *α*3*β*1, and *α*5*β*1) were upregulated under both MMF doses [41]. Dexamethasone (DEX), a glucocorticoid used commonly for topical ocular application, upregulated *α*v*β*3 integrin expression in the N27TM-2 cell line derived from human ocular trabecular meshwork, due to an increase of both the half-life and transcription of *β*3 integrin mRNA [42]. CsA also upregulated integrin *β*3 expression but in a dose-dependent manner, resulting in enhanced murine embryonic adhesion and invasion, which promoted embryo implantation [43]. Our work also showed an increase of *β*1 and *β*3 integrin expression in the presence of a therapeutic dose of Rapa and under CsA and Rapa combined (Figures 3 and 4). An earlier study demonstrated that integrins *β*1 and *β*3 mediate adhesion of murine CD8^+^ cytotoxic T lymphocytes (CTL) to fibronectin (FN), which increased signal triggering by an association of proline-rich tyrosine kinase-2 (Pyk2) with paxillin and the Src kinases, resulting in MHC I-peptide-driven CTL degranulation [48]. *β*1 and *β*3 integrins participate in adhesion of activated T cells to ECM proteins upon TCR triggering or, spontaneously, in secondary lymphoid organs or inflamed tissues, where they become highly exposed to ECM proteins [48, 49]. In this context it is difficult to explain the increase of *β*1 and *β*3 integrin expression we observed upon immunosuppressive drug treatment, but we note that the upregulation of integrin expression was accompanied by an increase of high-mannose/hybrid-type oligosaccharides on *β*1 and *β*3 integrins, as shown by the reaction with GNA (Figure 2(a)). The total surface expression of the high-mannose/hybrid-type N-glycans was also influenced by the immunosuppressive drugs we used; the combination of Rapa and CsA markedly increased the amount of those structures (Figure 1). Only a few previous studies have addressed the effect of immunosuppressive agents on leukocyte glycosylation. Paul et al. [46] showed that MMF inhibited IL-1-induced expression of GNA-recognized oligosaccharides with terminal mannose (Man) on rat endothelial cells. The ability of MMF to downregulate glycosylation results from MMF inhibition of inosine-monophosphate dehydrogenase, the enzyme that catalyses biosynthesis of (deoxy) guanosine nucleotides necessary for transfer of Man and fucose to glycoproteins [46, 50]. MMF-induced reduction of the expression and glycosylation of some adhesion molecules decreases the recruitment of lymphocytes and monocytes to sites of inflammation and graft rejection [50]. Itraconazole (Ita), one of the mTOR inhibitors, reduced poly-N-acetyllactosamine and tetra-antennary complex-type N-glycans in human umbilical vein endothelial cells (HUVEC) and caused an increase of Man5GlcNAc2 oligomannose structures on vascular endothelial growth factor receptor 2 (VEGFR2). Hypoglycosylation of VEGFR2 strongly inhibited its autophosphorylation after VEGF stimulation [15]. Induction of hypoglycosylation on VEGFR2 in HUVEC cells by Ita was similar to the effects of this drug in macrophages RAW 264.7. Glycosylphosphatidylinositol-anchored glycoprotein CD14 in RAW 264.7 became Endo H-sensitive in the presence of Ita. CD14 with altered glycosylation was delivered to the cell surface, as determined by binding of concanavalin A (Con-A) [51]. Alteration of glycan synthesis by immunosuppressive drugs has also been observed in cancer cells. Treatment of MDA-MB231 breast cancer cells with Rapa upregulated the sialylation of N-glycans on *β*1 integrin [45]. The clinically relevant concentration of CsA markedly decreased glucosylceramide levels in the multidrug-resistant human breast cancer MCF-7 cell line [52].

How does upregulation of GNA-positive glycoproteins, among the *β* integrins, driven by these anti-inflammatory immunosuppressive drugs, contribute to the development of immune tolerance against allogeneic antigens? We find a possible explanation in early studies by Muchmore et al. [13], which showed that mannose-rich oligosaccharide structures on uromodulin, ovalbumin, and soybean lectin (SBA) directly inhibit the antigen-driven T cell response in human mononuclear cell culture. What is more, treatment of endothelial cells with 1-deoxymannojirimycin (DMJ) and castanospermine (CAST), inhibitors which cause accumulation of high-mannose-type oligosaccharides, decreased interleukin 1-induced lymphocyte binding to endothelial cells; reduction of lymphocyte extravasation results in suppression of the immune response [53]. Similarly, the presence of immature high-mannose oligosaccharides on both subunits of FN receptor, integrin *α*5*β*1 in the cell surface of human fibroblasts, led to attenuation of ligand binding under treatment with DMJ. Inhibition of oligosaccharide processing at the high-mannose N-glycan stage did not alter receptor assembly and insertion into the plasma membrane, but it significantly modified integrin binding ability [14]. What is interesting is that the content of immature N-glycans diminishes during lymphocyte maturation; half of the N-glycans on CD45 on thymocytes are high-mannose or hybrid-type, while the majority of N-glycans on CD45 on peripheral T cells are ST6Gal1-modified complex-type structures [30]. Conversion of high-mannose to complex-type N-linked glycans is also necessary for differentiation of human B lymphocytes into cells secreting immunoglobulins (Ig), as determined by strong inhibition of Ig production in culture of human lymphocytes with DMJ or swainsonine (SW), which block conversion of high-mannose to complex-type glycans [54]. On the other hand, activation of alloantigen-reactive regulatory T cells (Treg) resulted in increased expression of *α*1,2-mannosidase, the enzyme that catalyzes removal of Man from Man9GlcNAc2 and generates Man5GlcNAc2. In mice, reduction of high-mannose structures was not required for the suppressive capacity of Treg but facilitated its migration to sites where it regulates the immune response [35]. The divergence of these results may indicate that the glycan-mediated effect is specific to the T cell type or organism. In view of the above-mentioned studies, we suggest that the abundant high-mannose/hybrid-type glycans on *β*1 and *β*3 integrins upregulated by CsA and Rapa may contribute to suppression of the immune response.

A variety of previous studies have shown that glycans play important roles in immune cell interactions during the immune response [5, 8, 33, 55], and now our results give further evidence that changes in oligosaccharide composition may shift the balance between the pro- and anti-inflammatory activities of leukocytes. Rapa and CsA have been shown to act synergistically for prolongation of allograft and xenograft survival [40, 47]. Possibly the changes in the glycosylation profile of leukocytes promote the development of tolerance in transplantation. New approaches aimed at developing better immunosuppressive strategies should incorporate the potential ability of drugs to alter glycosylation of target proteins.

## 5. Conclusions

Our study showed that these immunosuppressive drugs affect high-mannose/hybrid-type glycosylation of human leukocytes activated in an MLR reaction. CsA significantly reduced the number of leukocytes covered by high-mannose/hybrid N-glycans, and the synergistic action of CsA and Rapa led to an increase of these structures on the remaining leukocytes. This is the first study indicating that *β*1 and *β*3 integrins bearing high-mannose/hybrid structures are affected by Rapa and CsA. By regulating the protein amount, the immunosuppressive drugs increased the oligomannose/hybrid-type N-glycosylation on the leukocyte surface. This alteration of glycosylation on leukocytes may contribute to the development of immune tolerance. The functional consequences of changes in the glycosylation profile are an area for further study.

## Figures and Tables

**Table 1:** Cyclosporin A (CsA) and rapamycin- (Rapa-) affected GNA-positive glycoproteins identified by MS/MS. Proteins bearing high mannose/hybrid-type oligosaccharides expressed on MLR-activated leukocytes treated with a combination of CsA (150 ng per 1 mL medium) and Rapa (12 ng per 1 mL medium) were identified in MS/MS analysis by comparison of the results with the Sprot database. Ten glycoproteins with the highest protein score for each band are presented.

Band no.	Accession no.	Protein name	Mascot score	Number of significant peptide matches	Protein sequence coverage [%]	Mass based on amino acid sequence [kDa]	Relative mass calculated based on standard proteins [kDa]
	P05106	Integrin beta-3	6117	145	34	90.2	
	P14625	Endoplasmin	692	27	23	92.6	
	P05556	Integrin beta-1	618	19	16	91.7	
	Q9NZ08	Endoplasmic reticulum aminopeptidase 1	321	8	8	100.0	
	P19367	Hexokinase-1	315	7	7	100.0	
1	P11279	Lysosome-associated membrane glycoprotein 1	275	7	6	45.4	102.4
	O00462	Beta-mannosidase	236	9	9	101.8	
	P12259	Coagulation factor V	226	6	6	252.7	
	Q99467	CD180 antigen	205	2	2	75.2	
	Q8TB96	T-cell immunomodulatory protein	124	3	3	68.5	

	P05164	Myeloperoxidase	2491	88	25	83.8	
	P07237	Protein disulfide isomerase	502	18	14	57.1	
	P51688	N-Sulphoglucosamine sulphohydrolase	420	13	10	56.7	
	P16278	Beta-galactosidase	519	9	9	76.0	
2	P00488	Coagulation factor XIII A chain	581	14	14	83.2	60.5
	P01876	Ig alpha-1 chain C region	337	8	7	37.6	
	Q9Y4L1	Hypoxia upregulated protein 1	319	11	9	111.3	
	Q9NZK5	Adenosine deaminase CECR1	234	11	10	59.0	
	P08236	Beta-glucuronidase	208	6	5	74.7	
	Q9UHL4	Dipeptidyl peptidase 2	206	5	5	54.3	

	Q6P4A8	Phospholipase B-like 1	596	18	8	63.2	
	P15586	N-Acetylglucosamine-6-sulfatase	287	9	5	62.0	
	O14773	Tripeptidyl-peptidase 1	250	6	4	61.2	
	P01889	HLA class I histocompatibility antigen, B-7 alpha chain	172	3	3	40.4	
	Q9BS26	Endoplasmic reticulum resident protein 44	166	5	5	46.9	
3	Q07000	HLA class I histocompatibility antigen, Cw-15 alpha chain	121	3	3	40.8	42.6
	P05121	Plasminogen activator inhibitor 1	84	4	4	45.0	
	P04233	HLA class II histocompatibility antigen gamma chain	76	1	1	33.5	
	P10314	HLA class I histocompatibility antigen, A-32 alpha chain	73	3	3	41.0	
	P09326	CD48 antigen	67	2	2	27.7	
